# Parent-Child Agreement Using the Spence Children's Anxiety Scale and a Thermometer in Children with Autism Spectrum Disorder

**DOI:** 10.1155/2015/315495

**Published:** 2015-04-02

**Authors:** T. May, K. Cornish, N. J. Rinehart

**Affiliations:** ^1^Deakin Child Study Centre, Deakin University, Melbourne Burwood Campus, 221 Burwood Highway, Burwood, VIC 3125, Australia; ^2^School of Psychological Sciences and Monash Institute for Brain Development & Repair, Monash University, Clayton Campus, Building 17, Wellington Road, Clayton, VIC 3800, Australia; ^3^Deakin Child Study Centre, School of Psychology, Deakin University, Melbourne Burwood Campus, 221 Burwood Highway, Burwood, VIC 3125, Australia

## Abstract

Children with Autism Spectrum Disorder (ASD) experience high anxiety which often prompts clinical referral and requires intervention. This study aimed to compare parent and child reports on the Spence Children's Anxiety Scale (SCAS) and a child-reported “worry thermometer” in 88 children aged 8–13 years, 44 with ASD and 44 age, gender, and perceptual IQ matched typically developing children. There were no gender differences in child report on the SCAS and worry thermometers. Results indicated generally good correlations between parent and child self-reported SCAS symptoms for typically developing children but poor agreement in parent-child ASD dyads. The worry thermometer child-report did not reflect child or parent reports on the SCAS. Findings suggest 8–13-year-old children with ASD may have difficulties accurately reporting their anxiety levels. The clinical implications were discussed.

## 1. Introduction

Children with Autism Spectrum Disorder (ASD) consistently show high levels of anxiety. Around 40% of children and adolescents with ASD have clinically elevated anxiety levels or experience at least one anxiety disorder [1–4]. A recent meta-analysis of anxiety in ASD found that specific phobia was the most common subtype (30%), followed by Obsessive Compulsive Disorder (OCD; 17%), social anxiety disorder and agoraphobia (17%), generalized anxiety disorder (15%), separation anxiety disorder (9%), and panic disorder (2%) [3]. Anxiety related issues may prompt clinical referral and also require clinical intervention in this population. As children with ASD may quickly escalate their emotional states during “meltdowns,” assessing anxiety symptoms in a timely and valid manner is important for management at home, at school, and in clinical setting [5, 6]. A rapid assessment measure of anxiety in ASD would be useful in these contexts. This would allow parents, teachers, and clinicians to easily determine the level of anxiety in a child with ASD and, if elevated, employ an appropriate intervention. However, whether self-reported anxiety symptoms in children with ASD are valid is somewhat unclear.

A number of researchers have questioned the ability of children with ASD to self-report internal emotional states [7–10]. In empirical studies of ASD, there are mixed findings. Many have used the parent and child version of the Spence Children's Anxiety Scale (SCAS) in children with ASD. Russell and Sofronoff [11] investigated 10–13-year-old children with Asperger's Disorder compared to a clinically anxious normed group and found that parents of children with ASD rated their children as having higher levels of overall anxiety, obsessive compulsive symptoms, and specific phobias than parents of clinically anxious children. Children with ASD rated themselves as having similar levels of anxiety to clinically anxious children. Parents rated their children as having higher levels of separation anxiety, social phobia, and generalized anxiety than did their child, with child ratings significantly higher than parent ratings for obsessive compulsive symptoms. Magiati and colleagues [12] examined a nonreferred sample of children with ASD aged over 8 years (mean age 12 y 10 m) and found moderately good parent-child agreement for only three subscales (Physical Injuries, Generalised Anxiety Disorder, and separation anxiety). Farrugia and Hudson [13] found generally good parent-child correlations (*r* = .697) in 12–16-year-olds with Asperger's Disorder. Ozsivadjian et al. [14] also found good parent-child agreement on the total SCAS score in 10–16-year-old males with ASD. Potentially, the older child age may account for the better consistency between parent-child reports compared with younger children examined by Russell and Sofronoff and Magiati et al.

Other measures of anxiety have also been used to examine parent-child agreement on anxiety symptoms. Lopata and colleagues [15] found parents reported higher levels of anxiety than their 7–13-year-old children with ASD using the Behavior Assessment System for Children Second Edition. However, children with ASD self-reported similar levels of anxiety to comparison children. Parent and child anxiety symptoms showed poor correlations in the ASD group. White and colleagues [16] used the Multidimensional Anxiety Scale for Children, child and parent version in 12–17-year-olds with ASD. They also found child and parent reports were not significantly correlated. The validity of self-report measures in adolescents with ASD was questioned given only 23% self-reported clinically elevated anxiety scores despite all being diagnosed with an anxiety disorder. They noted that adolescents with ASD may underreport their anxiety, perhaps due to a lack of insight, because they have a different perspective about their own anxiety, or an unwillingness to truthfully report their difficulties. Using the Screen for Child Anxiety Related Emotional Disorders [17] in 8–14-year-olds with ASD, Blakeley-Smith et al. [18] found moderate interclass correlations between parent-child reports with parents reporting higher levels of anxiety than their children, except for separation anxiety. van Steensel and colleagues [19] compared child and parent reports also using the SCARED in 7–17-year-old children with ASD and an anxiety disordered group. Parent-child agreement on this instrument was poorer in the ASD group than in the anxiety disordered group.

Overall, there are equivocal findings, with some studies showing parents generally report higher levels of anxiety in their child with ASD compared with the child's own report [11, 15, 19], whereas other studies have found relatively good parent-child agreement [12–14, 16, 18]. These discrepancies may relate to different methodologies employed given various ages, diagnoses, gender proportions, measurement instruments, and child IQ levels used across studies. For example, a meta-analytic review of anxiety in ASD found age was associated positively with levels of Generalised Anxiety Disorder and negatively with Obsessive Compulsive Disorder (OCD) and separation anxiety [3]. The meta-analysis also found a complex relationship between type of ASD (Asperger's Disorder, PDD-NOS, or Autistic Disorder) and type of anxiety, including higher rates of Generalised Anxiety Disorder in Asperger's Disorder, higher OCD, and specific phobia in Autistic Disorder and lower rates of OCD in PDD-NOS.

The studies reviewed so far have examined parent-child agreement using multiple item questionnaires (such as on the SCAS). Given the cognitive and verbal deficits in this population, high levels of alexithymia, and difficulties answering open ended questions, more simple ways of assessing internal states are indicated [20]. Visual cues such as Visual Analogue Scales, for example, “emotional thermometers,” can be used to measure the strength of feelings [20]. Thermometer scales are frequently used in mood and anxiety interventions for individuals with ASD [21–23] and have the advantage of being a largely visual tool which is important in ASD where language delays and deviance are commonplace with relative strengths in visual skills.

Thermometer scales have long been used and validated in paediatric pain management to assess the level of pain [24–26] and emotional distress in hospital [27, 28] and nonhospital settings [29–31]. Generally, visual analogue pain scales show reasonably sound psychometrics [32]; however, there are some mixed findings. Some studies have shown poor agreement between parent and child ratings, with parents reporting generally lower levels of pain than their child [33]. Research suggests that children generally need to have normal IQ and be 7 years of age and older to use thermometer scales reliably [34].

The validity of these types of visual scales is largely unexamined in ASD. Lopata and colleagues [35] used a thermometer scale to examine stress in children aged 6–13 years with ASD. They found mild to moderate correlations between a stress thermometer scale and cortisol levels which was unexpected given the fact that child self-reports often do not correlate well with physiological measures [35, 36]. This indicated that children with ASD may have some capacity to rate their internal states accurately using a thermometer scale. No studies examining the validity of anxiety specific thermometers for ASD were found in the literature.

It is also noteworthy that studies in this area of ASD have generally failed to examine gender differences in parent-child agreement on anxiety symptoms. There are many more boys than girls diagnosed with ASD, yet how gender interacts with anxiety in ASD is not well explored. Girls from adolescence onwards are reported to experience higher levels of anxiety than boys [37], which may also be the case in ASD [38]. Younger girls with ASD may also show higher levels of social anxiety relative to boys with ASD, with this difference also reflected in typically developing children [39]. It is possible that girls and boys may also self-report different levels of anxiety, with this yet to be explored by gender in ASD.

The major aim of this study was to determine how well child and parent reports on the SCAS correlated in children with ASD. Secondly, the study aimed to determine if a “worry thermometer” correlated with the SCAS. We also sought to examine whether there were any gender differences in child reports on the SCAS and the “worry thermometer.” We investigated the following research questions. (1) How well do typically developing (TYP) and ASD children's self-reports correlate with their parent report on the SCAS? (2) How well do anxiety thermometer child-report ratings correlate with parent and child report on the SCAS and does this differ if a child has ASD? (3) Do boys and girls with ASD self-report similar levels of anxiety symptoms on the SCAS and a worry thermometer?

## 2. Method

### 2.1. Participants

In total, 88 children participated in this study. Participants with ASD were 44 children, 21 male and 23 female, with Autistic Disorder or Asperger's Disorder and aged between 8 and 13 years. This sample was taken from our larger study of gender differences in children with ASD, and hence females were oversampled compared to the normal male prevalence found in ASD [39]. Participants were recruited prior to the release of DSM-5. All clinical children were diagnosed using DSM-IV-TR criteria by registered psychologists and pediatricians prior to taking part in the study. The DSM-IV-TR criteria for Autistic Disorder or Asperger's Disorder were confirmed for ASD participants using our standard process involving reviewing diagnostic reports completed by pediatricians/psychologists and interviewing parents using a symptom checklist to ensure DSM-IV-TR criteria were fulfilled. In addition, all ASD participants were screened and confirmed to be within the clinical range on the Social Responsiveness Scale (SRS) parent report [40] which has demonstrated validity with the Autism Diagnostic Interview Revised [41]. To increase the diagnostic validity and homogeneity of the ASD sample, children with PDD-NOS were excluded given the fact that this condition is diagnosed only when there are subclinical autism symptoms or atypical presentation [42]. All participants were recruited through the Monash University Centre for Developmental Psychology and Psychiatry, the Autism Victoria “Get Involved” volunteer register, and private clinics in the Melbourne metropolitan area. Only children with a full-scale IQ of 70 and above were included in the study.

Forty-four typically developing children, 25 male and 19 female, who were matched based on gender, age, and perceptual IQ, were recruited from a Melbourne metropolitan primary school. These children were also aged between 8 and 13 years. None of these children had any prior history of parent or teacher reported developmental disability or psychopathology. Further, possible ASD symptoms in this population were screened with these children scoring within the typical range on the SRS parent report [40]. Children across both groups were excluded if they had a history of brain injury or any genetic disorders (such as Fragile X syndrome).

### 2.2. Measures

#### 2.2.1. Intellectual Functioning

For all children with ASD, intellectual ability was assessed using the Wechsler Intelligence Scale for Children IV (WISC-IV, [43]), Australian version. This yields a full-scale IQ, Verbal Comprehension Index, and a Perceptual Reasoning Index. The Wechsler Abbreviated Scale of Intelligence (WASI, [44]), which yields a full-scale IQ, a Verbal IQ, and a Performance IQ, was completed for all typically developing children. The typically developing children completed the WASI as it was a shorter assessment which reduced the time burden of their participation in the study. The WASI full-scale IQ is comparable to the WISC-IV full-scale IQ, the Verbal IQ comparable to the Verbal Comprehension Index of the WISC-IV, and the Performance IQ comparable to the Perceptual Reasoning Index from the WISC-IV [44].

#### 2.2.2. Parent- and Child-Reported Anxiety

The Spence Children's Anxiety Scale [45] parent report is a 38-item questionnaire based on DSM-IV-TR criteria for anxiety disorders in children. The child-report is a 45-item questionnaire which includes six positive filler items (i.e., “I am happy”) and one general item which are not scored in either the total or subscale scores. Hence, the child report has 38 items which are scored and correspond directly with the parent version. The scale assesses six domains of anxiety including generalized anxiety, panic/agoraphobia, social phobia, separation anxiety, Obsessive Compulsive Disorder, and Physical Injury Fears. Parents and children report how often each of the items happens to their child using a four-point scale: “never,” “sometimes,” “often,” and “always.” Validity and reliability have been established [45–47]. The SCAS has previously been employed and shown to be a valid measure of anxiety levels in individuals with ASD [11, 13]. This measure does not have a unitary cutoff point but employs age and gender based norms to place children in clinically significant or the normal range of anxiety.

This study also used a 100-point Visual Analogue Scale (VAS) for the assessment of self-reported anxiety (worry thermometer adapted from [48]). Higher scores on this scale reflected higher levels of anxiety. The thermometer was presented on an A4 sized page with the thermometer presented vertically in the middle of the page marked from 0 (bottom) to 100 (top) with marks at 1-point intervals. A score of 0 indicated “Not at all worried,” 50 “worried,” and 100 “very very worried,” with these words placed next to their corresponding point on the scale. Three worry thermometers were utilised which referred to current worry, the worst worries in the last 2 weeks, and the worst worries in the child's lifetime. Children are asked for the two-week thermometer, “Including the last two weeks, what's the most worried/scared you've ever felt?”; for the ever thermometer, “What is the most worried/scared you've ever felt?”; and the now thermometer, “How worried/scared do you feel now?”

### 2.3. Procedure

The study was approved by Human Research Ethics Committees of Monash University and the Victorian Government Department of Education and Early Childhood. Parents received an explanatory statement and provided written informed consent. Children provided assent. Participation was voluntary and participants did not receive any monetary reward for participation other than reimbursement for travel costs.

Parents of participants were invited to participate via email or letter and follow-up telephone call. Participants were tested at a home visit, at the Monash University campus, or at their primary school. The WASI and WISC-IV were administered according to standardized instructions.

Parents filled out the questionnaires as per their standard instructions. Age-based standardized scores were utilized for the WISC-IV and WASI. Raw scores were used in analyses unless otherwise stated. Children completed the SCAS and worry thermometers in the presence of the examiner (TM) who explained and clarified any questions about the measures and read the questions from the SCAS for all children. All data were entered into statistical package for the social sciences (SPSS) version 22.0 for statistical analyses.

## 3. Results

### 3.1. Preliminary Analyses

Analyses utilised included *t*-tests to compare demographic variables between groups and mixed model analysis of variance (ANOVA) to compare group differences in anxiety measures across raters and gender. Pearson correlations were used to determine the associations between variables. Bonferroni corrections were employed for post hoc tests.

Independent *t*-tests showed no difference in age *t*(86) = −.440, *P* = .661 and perceptual IQ *t*(86) = 1.436, *P* = .155, between the ASD and TYP groups, Table 1. Children with ASD had lower Verbal IQs than TYP children, *t*(86) = 2.573, *P* = .012. The group was matched on gender according to a Chi square test,  *χ* ^2^(88) = .729, *P* = .393.

### 3.2. Group and Gender Differences on the SCAS

**Figure 1:**
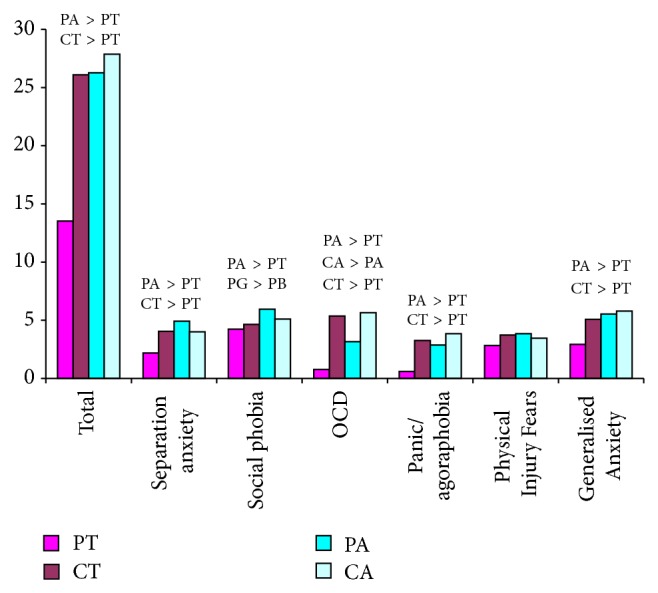
Means of the SCAS total and subscales for parents and children by group. Significant group differences are coded as follows: PA, parents of children with ASD; PT, parents of typically developing children; CA, children with ASD; CT, typically developing children; PG, parents of girls; PB, parents of boys.

The means of the SCAS total score and subscales by parent, child, and group are found in Figure 1, with group differences described in the following analyses. For the SCAS total score, a Repeated Measures ANOVA with group (ASD or TYP) and gender (male or female child) as the between-subject factors and SCAS rater (parent or child) as the repeated measure was conducted. This showed a significant main effect of group, *F*(1,84) = 11.978, *P* = .001, *η*_*P*_^2^ = .125, and rater, *F*(1,84) = 19.132, *P* = .001, *η*_*P*_^2^ = .186. There was a significant group by rater interaction, *F*(1,84) = 11.653, *P* = .001, *η*_*P*_^2^ = .122. To explore the interaction, independent *t*-tests were used with Bonferroni corrections (.05/4 = .0125). These found no difference between TYP and ASD child self-reports, *t*(86) = −.569, *P* = .517, but parents of children with ASD reported higher levels of anxiety than parents of TYP children, *t*(86) = −7.023, *P* < .001. Paired samples *t*-test showed there was no difference between parent and child reporting total anxiety for children with ASD, *t*(43) = −.654, *P* < .517, but there was a difference for the TYP group with TYP children reporting higher levels of anxiety than their parents, *t*(43) = −6.252, *P* < .001.

The six subscales were then explored using a Repeated Measures ANOVA to compare rater (parent versus child), group (TYP versus ASD), and gender (male versus female child). There was a significant main effect of subscale, *F*(1,84) = 24.573, *P* < .001, *η*_*P*_^2^ = .226, rater *F*(1,84) = 19.132, *P* < .001, *η*_*P*_^2^ = .186, and group, *F*(1,84) = 11.978, *P* < .001, *η*_*P*_^2^ = .125. There was a significant interaction between rater and group, *F*(1,84) = 11.653, *P* = .001, *η*_*P*_^2^ = .122, and subscale and rater *F*(1,84) = 21.811, *P* < .001, *η*_*P*_^2^ = .206. These were explored using post hoc ANOVAs with Bonferroni corrections for each subscale (.05/4 = .0125) and the differences are summarised in Figure 1. For Physical Injury Fears, there were no significant differences between parent-child reports.

For separation anxiety, there was a significant difference in parent reports (*P* < .001) for TYP and ASD children. TYP parents reported lower levels of separation anxiety than their children (*P* < .001) whereas in ASD parent-child dyads levels were similar.

For social phobia, parent-child dyads reported similar levels regardless of diagnosis. There was a significant difference between TYP and ASD parent reports (*P* = .007) with ASD parents reporting higher levels, but no difference between child reports.

Similarly, for Obsessive Compulsive Disorder, parents of children with ASD reported higher levels than parents of TYP children (*P* < .001) but there was no difference in child report. Both parents of children with ASD (*P* < .001) and parents of TYP children (*P* < .001) reported lower levels of OCD than their children.

Parents of TYP children reported significantly lower levels of panic/agoraphobia than their children (*P* < .001), with no difference between ASD parent-child reports. Parents of children with ASD reported higher levels of panic/agoraphobia than parents of TYP children (*P* < .001), with children reporting similar levels.

For Generalised Anxiety, parents of TYP children reported lower levels than their children (*P* < .001), with no difference between ASD parent-child dyads. Parents of children with ASD reported higher levels than parents of TYP children, with no difference in self-reported Generalised Anxiety in children with or without ASD.

For gender there was only one subscale by gender interaction, *F*(1,84) = 2.391, *P* = .037, *η*_*P*_^2^ = .028, with a significant difference for parent-reported social phobia (*P* < .001), with inspection of means revealing that girls were reported to experience higher levels (*M* = 6.26, SD = 3.22) than boys (*M* = 4.02, SD = 2.41). There were no other gender differences in any other analyses, including the thermometer scales.

### 3.3. Child Thermometer Scales

Pearson correlations between the now, ever, and two-week scales were calculated for the TYP and ASD groups. For the TYP group, the ever and two-week scales were correlated, *r* = .459, *P* = .002, but the now and ever (*r* = .151, *P* = .328) and now and two-week scales (*r* = .280, *P* = .065) were not. For the ASD group, the same pattern was present. The ever and two-week scales were correlated, *r* = .304, *P* = .045, but the now and ever (*r* = .144, *P* = .352) and now and two-week scales (*r* = .199, *P* = .196) were not.

An ANOVA showed no group differences in the child-reported anxiety thermometer scales (now, two weeks, ever) in regard to ASD (two weeks *M* = 35, SD = 33; now *M* = 11, SD = 21; ever *M* = 79, SD = 76) versus TYP children (two weeks *M* = 29, SD = 21; now *M* = 5, SD = 10; ever *M* = 72, SD = 26). Given the fact that Verbal IQ was correlated with the 2-week thermometer, an ANCOVA was conducted and similarly found no difference between TYP and ASD children.

### 3.4. SCAS Parent and Child Correlations

Correlations between the SCAS parent and child reports for both groups are shown in Table 2. In children with ASD, there was a significant correlation between parent- and child-reported separation anxiety. In TYP children, there were significant correlations between parent- and child-reported separation anxiety, social phobia, panic/agoraphobia, Physical Injury Fears, and the SCAS total score.

### 3.5. Child Thermometer Correlations with Parent and Child SCAS Scores

Pearson correlations between the child thermometers and parent and child SCAS scores were calculated. For child reports on the SCAS and thermometer, in the ASD group, separation anxiety (*r* = .380, *P* < .05), social phobia (*r* = .441, *P* < .01), and the SCAS total score (*r* = .305, *P* < .05) correlated with the ever thermometer; Physical Injury Fears (*r* = .373, *P* < .05) with the now thermometer; and social phobia (*r* = .304, *P* < .05), panic/agoraphobia (*r* = .357, *P* < .05), and Generalised Anxiety (*r* = .319, *P* < .05) with the two-week thermometer. In TYP children, there was only one correlation which was between separation anxiety and the now thermometer (*r* = .350, *P* < .05).

In regard to parent-child agreement, in the TYP group, the two-week thermometer was unexpectedly negatively correlated with parent-reported separation anxiety (*r* = −.327, *P* < .05). For children with ASD, the now thermometer was correlated with parent-reported OCD (*r* = .309, *P* < .05) and Generalised Anxiety (*r* = .352, *P* < .05). The lack of associations indicated that the thermometer was not measuring the same anxiety construct as the SCAS.

## 4. Discussion

In the present study, we sought to compare parent and child reports of child anxiety using the SCAS in children with ASD and typically developing children aged 8–13 years. We found that typically developing parent-child reports on the SCAS showed good correlations, whereas ASD parent-child reports were generally poorly correlated. We also examined whether a “worry thermometer” was able to measure anxiety and found poor construct validity with the SCAS.

### 4.1. TYP versus ASD on the SCAS

Children with ASD rated themselves as having similar levels of anxiety to typically developing children across all subscales and for the total score, which was in contrast to some past studies [11] but consistent with others [15]. This was despite ASD parents reporting significantly higher levels of anxiety in regard to total anxiety, social phobia, separation anxiety, OCD, and Generalised Anxiety than did TYP parents, a finding consistent with some past studies [15, 18]. Hence, even though children with ASD have high levels of anxiety according to parent report, they self-report similar levels to typically developing children. This may relate to poorer emotion recognition in children with ASD who may have difficulty recognizing and expressing their anxiety, despite them being cognitively high-functioning in the present study. For example, although children with Asperger's Disorder generally have superficially normal language development, their comprehension may be particularly poor [49] which may impact on their ability to understand and express complex constructs such as their own anxiety. Research in adults with ASD suggests up to 50% have alexithymia which impacts on the ability to recognize and express emotional states [50, 51].

Parents of TYP children reported lower levels of anxiety than did their children with the exception of Physical Injury Fears and social phobia. These types of anxiety may be more readily observed by parents in child behavior, potentially resulting in better concordance on these two measures. ASD parents reported lower levels of OCD than their children, with otherwise similar mean scores for the other five subscales and total SCAS score. Russell and Sofronoff [11] similarly found that children with ASD rated themselves as having higher levels of OCD than their parents indicated. Overall, ASD parents and children actually reported more similar mean levels of anxiety, with TYP parents generally reporting lower mean levels of anxiety than their children. It may be that TYP children overreport their levels of anxiety and children with ASD underreport theirs, resulting in this pattern of similar child-reported anxiety levels.

### 4.2. Correlations between Parent and Child SCAS

Although at the group level there was general consistency between mean parent- and child-reported levels of anxiety, the parent-child* correlations* suggested poor agreement in ASD parent-child dyads compared with better agreement in TYP parent-child dyads. These findings are in contrast to some past studies [12–14], which found generally good child-parent correlations on the total SCAS score in youth with ASD, but consistent with White and colleagues [16] who found poor parent-child correlations (12–17 years). These differences may be due to the older children in these three aforementioned studies (10–16 years) compared to the current study (8–13 years). The SCAS may be better able to accurately capture self-reported anxiety in older children with ASD. Consistent with the current study, Lopata and colleagues [15] and van Steensel and colleagues [19] also found poor correlations between parent-child-reported anxieties in children with ASD using other anxiety measures. Their age ranges also included younger participants (7–17) further supporting the notion that age may be a factor in the accurate self-reporting of anxiety in ASD.

### 4.3. Thermometer Scale

This is the first study to our knowledge to compare a Visual Analogue Scale to the SCAS in children with ASD. The child-rated worry thermometer used in the study showed poor construct validity with parent and child reports on the SCAS for both TYP and ASD groups. There have been some studies which have not found Visual Analogue Scales to be well correlated with parent reports, at least in the paediatric pain literature [33]. Parent-child agreement is good for severe difficulties (such as severe injuries and things complained of daily), but less for more infrequent or less severe difficulties [52]. The thermometer scale used in the current study asked children to report on how “worried” or “scared” they were, which can be considered two separate constructs; hence, this could explain the lack of validity of the thermometer. Future work may benefit from separating these constructs into separate thermometers.

### 4.4. Gender

Finally, this was the first study to compare self-reported anxiety in girls and boys with ASD. There were no gender differences found in boys and girls ratings of their own anxiety in the ASD or TYP groups, on both the SCAS and worry thermometers. Potentially, as girls reach puberty, they may experience greater levels of anxiety than boys and show differences in anxiety self-report from this period onwards.

### 4.5. Clinical Implications

The present findings contribute to a growing body of research showing that children with ASD prior to puberty may have difficulties accurately reporting their anxiety. This is not the case for typically developing children which may relate to their better verbal and emotional understanding. Parent report, at least using the SCAS, is likely to be a more accurate measure of anxiety than children with ASD's self-report. Hence, clinicians and those who work with children with ASD within the 8–13-year age range may need to primarily rely on parent reports of anxiety. Additionally, the use of thermometer scales to measure internal states in children with ASD is commonplace in a number of ASD specific interventions [20, 22, 23]. Clinicians should interpret these child self-reported thermometer scales cautiously until validity is demonstrated.

### 4.6. Limitations

An important extension of this study will be for clinicians to rate anxiety in the children using structured parent and child interviews. In the present study, the child ratings were compared to their parents and not to a clinical assessment of anxiety symptoms. This will then allow clinician reports to be correlated with child and parent reports of anxiety to further examine the validity of a thermometer scale. The lack of a clinician report is a major limitation of the study. However, it is noteworthy that past studies that have compared parent, child, and clinician structured interviews in youth with ASD have found good to excellent correspondence between clinician consensus diagnosis and parent reports of anxiety [53]. Comparing a group of children with anxiety disorders without ASD to those with ASD on the thermometer scale may have also been informative to determine whether the poor correspondence between parent-child reports is associated with anxiety per se or ASD.

Only children with full-scale IQs greater than 70 were included, and hence these findings may not generalize to children with ASD and intellectual disability. Of note, none of the studies examining self-reported anxiety in ASD discussed have included children with intellectual disability as participants. Given the fact that most individuals with ASD have intellectual disability, examining whether similar self-report measures of anxiety are valid in this population is needed.

## 5. Conclusion

Overall, children with ASD aged between 8 and 13 years show difficulty in accurately reporting their anxiety relative to parent reports. In contrast, typically developing children of this age range self-report anxiety levels similar to their parent ratings. The use of a visual analogue “worry thermometer” to gauge anxiety levels in children with ASD was not supported by the present studies findings. Given the extensive use of these types of scales in interventions for children with ASD, further research into the validity of these measures is warranted.

## Figures and Tables

**Table 1:** Demographic characteristics of the Autism Spectrum Disorder and typically developing groups.

Variable	ASD group (*N* = 44)	TYP group (*N* = 44)
Age in months M (SD)	124.7 (19.3)	122.9 (19.5)
Boys : girls	21 : 23	25 : 19
Verbal IQ M (SD)	100.7 (13.2)	107.1 (9.6)
Performance IQ M (SD)	100.7 (15.3)	105.2 (13.9)

ASD: Autism Spectrum Disorder; TYP: typically developing.

**Table 2:** Agreement between parent and child SCAS subscales and total score for the ASD and TYP groups.

	Child SA	Child SP	Child OCD	Child PA	Child PIF	Child GA	Child total
Typically developing							
Parent Separation Anxiety	.299^*^	.112	.262	.319^*^	.018	.084	.262
Parent Social Phobia	.067	.336^*^	−.031	−.036	.247	−.122	.100
Parent Obsessive Compulsive Disorder	.236	.117	.083	.068	.122	−.059	.130
Parent Panic/Agoraphobia	.295	.291	.155	.343^*^	.250	.166	.342^*^
Parent Physical Injury Fears	.020	.327^*^	−.142	−.042	.517^**^	.222	.171
Parent Generalised Anxiety	.238	.130	.195	.282	.354^*^	.213	.315^*^

Parent total	.268	.360^*^	.113	.206	.392^**^	.104	.320^*^

ASD							
Parent Separation Anxiety	.306^*^	.345^*^	.049	.219	.220	.223	.287
Parent Social Phobia	−.037	.180	−.167	−.033	.052	.001	−.005
Parent Obsessive Compulsive Disorder	−.016	.056	.106	.085	.105	.128	.100
Parent Panic/Agoraphobia	.223	.322^*^	.058	.145	.169	.232	.244
Parent Physical Injury Fears	.121	.053	−.026	.036	.290	.012	.089
Parent Generalised Anxiety	.248	.218	−.007	.168	.219	.196	.215

Parent total	.227	.324^*^	−.006	.163	.280	.209	.247

SA, separation anxiety; SP, social phobia; OCD, Obsessive Compulsive Disorder; PA, panic/agoraphobia; PIF, Physical Injury Fears; GA, Generalised Anxiety, SCAS, Spence Children's Anxiety Scale; ^*^ *P* < .05, ^**^ *P* < .01.
